# 

**DOI:** 10.1192/bjb.2019.6

**Published:** 2019-04

**Authors:** Sebastian Kraemer

**Affiliations:** Honorary Consultant Psychiatrist, Tavistock and Portman NHS Trust, UK. Email: kraemer@doctors.org.uk

‘I do not consider precision in diagnosis, or even assessment, to be the main goal. I want us to remember that helping a child forward is the main goal.’ Andrew West's learned lament for what has been forgotten about the primary task of child and adolescent psychiatry is required reading for anyone engaged in child mental health. Often ironic, his text puts us in the position of a child visiting a Child and Adolescent Mental Health Services (CAMHS) clinic: ‘the child will be looking for some genuineness … children are as acutely aware of bullshit as they are of condescension and they are not fooled’. Here is a doctor who sees it as his duty to identify with his patient: ‘I know what it feels like to be misunderstood,’ says Andrew, taking the further step to see ‘that it may not be in [the child's] greater interests to be free of this particular symptom’. That symptoms have functions is a sophisticated perspective, encouraging the clinician not to hurry too quickly to remove them.

Andrew cites the great paediatrician-turned-psychoanalyst Donald Winnicott's view that it is necessary to ‘contain the conflicts … rather than anxiously looking around for a cure’ (p. 2).^1^ In that spirit – though it may only be for a short time – the psychiatrist offers himself as a ‘powerful therapeutic companion’ who is ‘able to slow time down and actually enable treatment to take place’. This creative encounter is not likely when the clinical service has a very limited number of answers. ‘A parent says, “I want my child to behave better,” and the clinician says, “this is ADHD; I suggest that you attend a parenting group and I can prescribe medication for your child”. It is reminiscent of a conversation between two deaf people along the lines of, “are you thirsty? No, it's Friday”.’

Since diagnostic psychiatry became the dominant mode around three decades ago, too little attention has been paid to consultative skills in the profession. Andrew West proposes an ethical practice for modern child and adolescent psychiatrists, placing particular emphasis on the initial assessment and on the ‘saying goodbye’, both of which can be powerfully therapeutic in themselves, whatever other clinical intervention may be provided. Dr West obliges us to see the experience from the patient's and family's point of view, where ‘treatment’ is not just what is ordered after an assessment but something that ‘begins at the point that the service accepts the referral’. A beautifully written, highly intelligent and inspiring book.
